# Catamenial pneumothorax with partial liver herniation due to diaphragmatic laceration: a case report and literature review

**DOI:** 10.1186/s13019-021-01407-z

**Published:** 2021-03-17

**Authors:** Satoshi Arakawa, Hideki Matsudaira, Yuki Noda, Makoto Yamashita, Jun Hirano, Masaichi Ogawa, Takashi Ohtsuka

**Affiliations:** 1 Department of Surgery, The Jikei Katsushika Medical Center, 6-41-2, Aoto, Katsushika-ku, Tokyo, Japan; 2 Division of Thoracic Surgery, Department of Surgery, The Jikei University School of Medicine, 3-25-8, Nihi-shinbashi, Minato-ku, Tokyo, Japan

**Keywords:** Catamenial pneumothorax, Diaphragmatic defect, Liver herniation, Endometrial tissue

## Abstract

**Background:**

Catamenial pneumothorax is generally uncommon, with an incidence of less than 3–6% in women with spontaneous pneumothorax. As few cases of catamenial pneumothorax with diaphragmatic defect and liver herniation have been reported, this case report may be useful for understanding the cause and treatment. This case highlights the importance of the approach for liver hernia in patients with catamenial pneumothorax and endometriosis.

**Case presentation:**

We report a case of catamenial pneumothorax in a 43-year-old woman with diaphragmatic partial liver hernia who was treated with thoracoscopic surgery. She was diagnosed with a right pneumothorax at menstruation onset. Chest computed tomography showed a nodule protruding above the right diaphragm. We performed thoracoscopic surgery to treat the persistent air leak and biopsied the nodule on the right diaphragm. There were blueberry spots on the diaphragm; the nodule was found to be the herniated liver. The diaphragmatic defect was sutured. Histological examination of the tissue near the partial prolapsed liver revealed endometrial tissue.

**Conclusions:**

It is speculated that ectopic endometrial tissue in the diaphragm will periodically necrose to become a diaphragmatic tear, which is a pathway for air to enter the thoracic cavity and eventually a herniated liver. Thoracoscopic surgery should be considered in patients with catamenial pneumothorax when a diaphragmatic lesion is suspected.

## Background

Catamenial pneumothorax (CP) is generally uncommon, with an incidence of less than 3–6% in women with spontaneous pneumothorax [1]. Only a few cases of CP with diaphragmatic defect and liver herniation have been reported. Furthermore, clinical or pathological endometriosis is found in only 22–37% of patients [2]. We report a case of CP in a patient with a diaphragmatic partial liver hernia who was treated with thoracoscopic surgery. Endometrial tissue was found in the thoracoscopic diaphragmatic resection specimen. Few cases of catamenial pneumothorax with diaphragmatic defect and liver herniation have been reported; therefore, this case report may be useful for understanding the cause and treatment. This case highlights the importance of the approach for liver hernia in patients with catamenial pneumothorax and endometriosis. A review of previous reports is also provided.

## Case presentation

We report this case in accordance with the ethical standards of the Ethics Committee of Jikei University School of Medicine (date: February 12, 2020, approval number: 31–384(9964)) and the Declaration of Helsinki and its later amendments. The need for individual patient consent was waived due to the retrospective design of the case report.

**Fig. 1:**
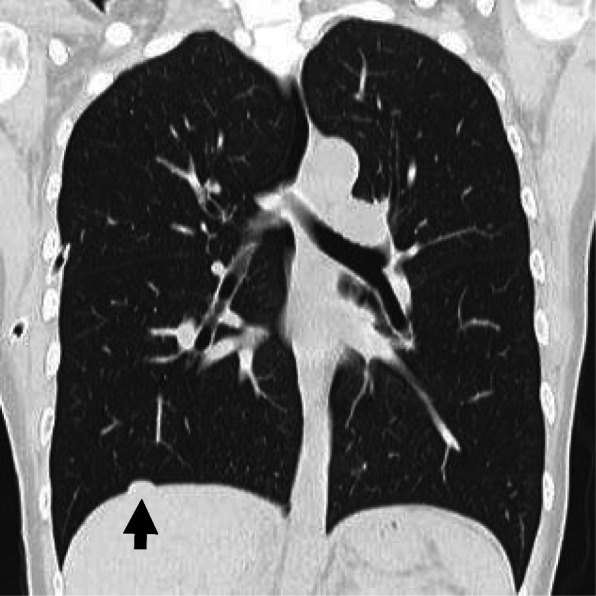
Chest CT scan showing a nodule (arrow) on the right diaphragm without bullae or blebs

**Fig. 2:**
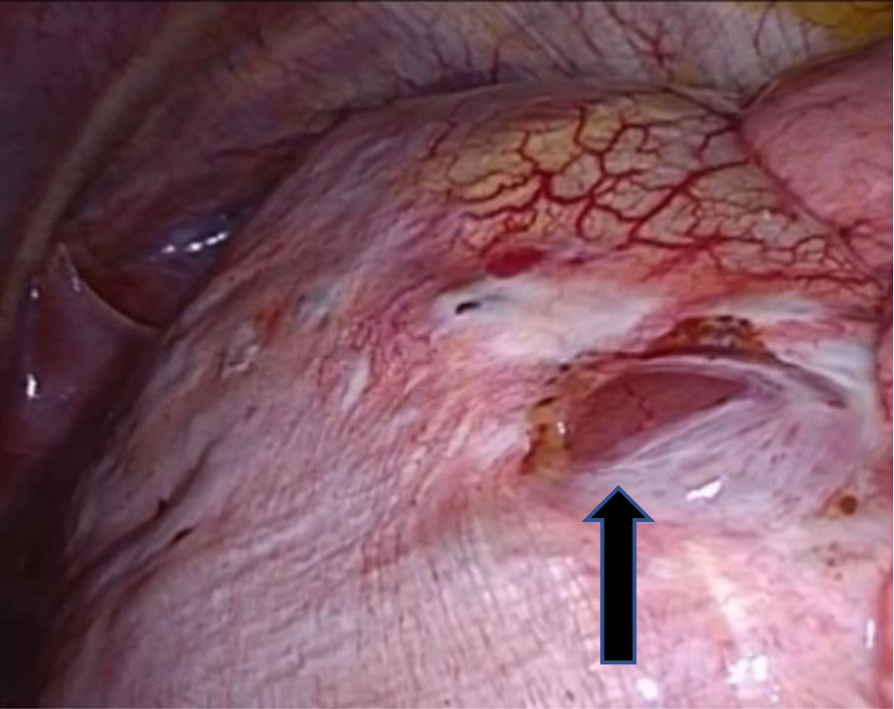
Thoracoscopic view of the liver herniating into the thoracic cavity through a diaphragmatic defect (arrow)

**Fig. 3:**
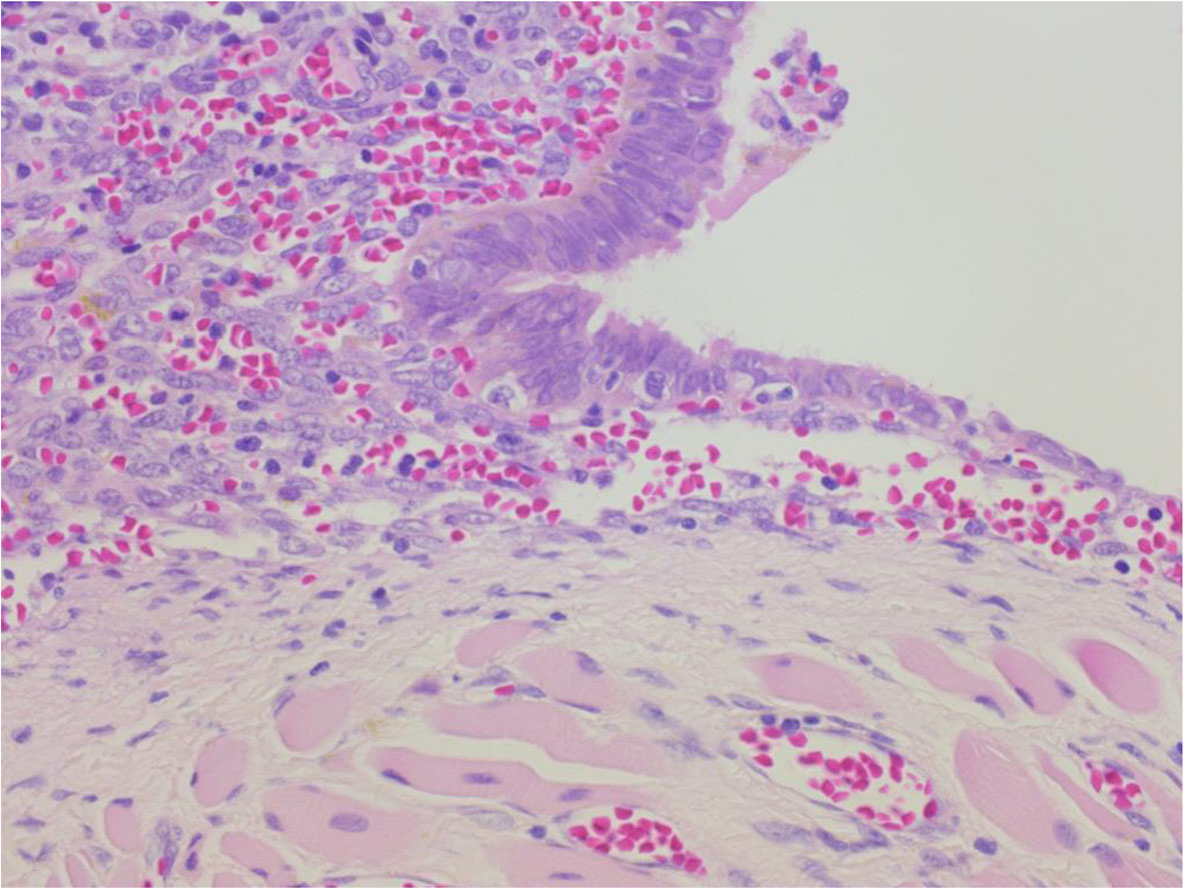
Microscopic findings of the resected diaphragm (hematoxylin and eosin staining, × 100)

The patient was a 43-year-old woman who presented to the Jikei Katsushika Medical Center complaining of right chest pain. She had suffered from two previous right pneumothoraces (August 2015 and May 2016) and had a history of endometriosis. She was admitted to our hospital in December 2017 with a third right pneumothorax. There was no history of chronic medication use or smoking, and blood biochemical findings, including tumor markers, were unremarkable. The onset of the pneumothorax occurred shortly after the onset of menstruation, and preoperative chest computed tomography revealed no obvious bullae or blebs and a nodule of approximately 1 cm in the right diaphragm (Fig. 1). The classification of pneumothorax was suspected to be CP, and the diaphragmatic nodule was expected to be ectopic tissue of the endometrium. Thoracoscopic surgery was performed due to a persistent air leak and to obtain a biopsy specimen of the diaphragmatic nodule. The operation was initiated with general anesthesia, differential lung ventilation, and the patient in a left lateral supine position. We performed video-assisted thoracic surgery (VATS) in 3 ports (4th intercostal anterior axillary line, 5th intercostal posterior axillary line, and 9th intercostal mid-axillary line). There were scattered blueberry spots on the diaphragm, and the right diaphragmatic nodule was found to be liver tissue that prolapsed into the thoracic cavity through a diaphragmatic defect of less than 1 cm (Fig. 2). There was no apparent responsible lesion in the lung, and the mediastinal pleural fat, a possible ectopic endometrial area on the diaphragm, and the blueberry spot were resected. The diaphragmatic defect was sutured (2–0 Prolene®) and closed after adhesive dissection of the liver. Absorbent tissue reinforcement (NEOVEIL® sheet) and fibrin sealant (Beriplast®) were used to cover the entire diaphragm. The operative time was 3 h and 8 min, and the volume of blood loss was 3 mL. The chest tube was removed on the second postoperative day after confirming that there was no air leak, and the patient was discharged on postoperative day three. Histopathological findings (Hematoxylin-Eosin stain) showed abundant interstitial tissue in contact with the diaphragm, glandular structures, and hemorrhage in the interstitium (Fig. 3). These findings led to a diagnosis of ectopic endometriotic tissue. As of April 2020 (two years and four months post-operatively), she was continuing luteal hormone therapy (dienogest 2 mg/day) and has had no recurrence of pneumothorax or diaphragmatic hernia.

## Discussion and conclusions

The characteristics of CP with menstruation include (1) repetitive onset of pneumothorax before and after menstruation, (2) more common in the 30- to 50-year age range, (3) right-sided onset, (4) no onset observed during menopause, pregnancy, or after menopause, (5) the onset of pneumothorax and reduction of symptoms can be prevented with ovulation suppressants, and (6) pelvic endometriosis is present in many cases [3, 4]. The following three general mechanisms have been proposed for the development of CP: “migration theory,” “metastatic or lymphovascular microembolization theory,” and “physiologic hypothesis” [2].

A search for “catamenial pneumothorax” AND “diaphragmatic hernia” in PubMed retrieved 14 reports from 1992 to 2019 (Table 1). Ten patients had hernias on the right side, the mean age was 40.6 years (35–45 years), and nine underwent surgery. Multiple defects characterized the hernias, the size of the hernia orifice ranged from 3.3 cm to 10 cm, and the content of the diaphragmatic hernia was the liver in all cases. The treatment strategies consisted of VATS (4 cases), open thoracotomy (4 cases), and open thoracotomy + laparotomy (1 case). The treatment strategies for the remaining five patients are unknown. Suture repair with non-absorbable sutures was performed in 2 cases, suture repair with non-absorbable sutures + pleurodesis therapy was performed in 1 case, suture repair with non-absorbable sutures + pleurodesis therapy + synthetic mesh coating in 1 case, suture repair with non-absorbable sutures + pericardial patch + pleural abrasion in 1 case, pleural abrasion + pleural adhesion therapy in 1 case, pericardial patch in 1 case, and pleural abrasion + mesh coating in 1 case.

**Table 1:** Surgical cases of diaphragmatic hernia associated with paramenstrual pneumothorax reported in the literature

Reference	Age	Diagnisis	Symptoms	bulla	Size of defect (cm)	Herniation	Pathology	Surgical treatment	Medical treatment	Outcome
Bobbio et al. 2007 [5]	35	Right recurrent pneumothorax	Dysmenorrhoea	No	4	Liver	–	Primary closure; pleurodesis	–	–
Bostoen et al. 2011 [6]	37	Pneumothorax	Left iliac fossa pain	–	–	Liver	–	Plication; pleurodesis	–	–
Sanna et al. 2011 [7]	38	Right recurrent pneumothorax	–	–	Not mentioned (“Multiple defects”)	Liver	–	Plication; pleurodesis	–	No recurrence at 4 months
Tomescot et al. 2012 [8]	45	Right recurrent pneumothorax	–	–	Not mentioned (“Multiple defects”)	Liver	No endometriosis	Patch repair	–	No recurrence at 18 months
Visouli AN et al. 2012 [2]	38	Right pneumothorax	–	–	Not mentioned (“Multiple defects”)	Liver	–	Patch repair	Ovarian suppression treatment	Well and asymptomatic upon follow-up
Yu & Sihoe 2015 [9]	44	Right pneumothorax	–	Yes	10	Liver	Endometriosis	Mesh repair; pleurodesis	Ovarian suppression treatment	No recurrence at 15 months
Ashwad et al. 2016 [10]	39	Right pneumothorax	Chest pain	–	Not mentioned	Liver	Endometriosis	Primary closure talc pleurodesis	–	–
Demetrio et al. 2018 [11]	42	Right recurrent pneumothorax	Chest pain	No	–	Liver	Endometriosis	Primary closure;	–	No recurrence at 5 months
Mukku VK et al. 2019 [12]	40	Right pneumothorax	Chest tightness	No	3.3	Liver	Endometriosis	Mechanical pleurodesis, Primary closure	–	No recurrence at 2 months
Current case	43	Right pneumothorax	Chest pain	No	1	Liver	Endometriosis	Primary closure Absorbent tissue reinforcement and fibrin sealant	Ovarian suppression treatment	No recurrence at 28 months

In the present case, the patient had a history of endometriosis, and a diaphragmatic hernia was confirmed on the basis of the intraoperative findings. The presence of endometrial tissue was confirmed upon pathological examination of the diaphragm, and there was no recurrence with closure of the diaphragmatic defect and hormone therapy. There was no blueberry spot on the visceral side of the pleura to suggest the presence of endometrial tissue or obvious emphysematous changes, and it is most likely that air entered the thoracic cavity via a diaphragmatic defect and caused a pneumothorax. It is speculated that ectopic endometrial tissue in the diaphragm will periodically necrose to become a diaphragmatic tear and eventually a herniated liver [2, 13].

This case highlighted the approach for treatment of liver hernia in patients with catamenial pneumothorax and endometriosis. It also showed that repairing the hernia and using hormone therapy were able to prevent recurrence, thus offering a suggestion for an effective treatment strategy. Further, to the best of our knowledge, very few such cases have been reported, which increases the usefulness of this case report with regard to understanding treatment options. Thoracoscopic surgery should be considered in patients with CP when a diaphragmatic lesion is suspected.

## Data Availability

All data in the current report are not publicly available due to personal privacy but are available from the corresponding author upon reasonable request.

## Abbreviations

CP: Catamenial pneumothorax
VATS: Video-assisted thoracic surgery

## References

[1] Blanco S, Hernando F, Gómez A, González MJ, Torres AJ, Balibrea JL (1998). Catamenial pneumothorax caused by diaphragmatic endometriosis. J Thorac Cardiovasc Surg.

[2] Visouli AN, Zarogoulidis K, Kougioumtzi I, Huang H, Li Q, Dryllis G, Kioumis I, Pitsiou G, Machairiotis N, Katsikogiannis N, Papaiwannou A, Lampaki S, Zaric B, Branislav P, Porpodis K, Zarogoulidis P (2014). Catamenial pneumothorax. J Thorac Dis.

[3] Maurer ER, Schaal JA, Mendez FL (1958). Chronic recurring spontaneous pneumothorax due to endometriosis of the diaphragm. JAMA..

[4] Lillington GA, Mitchell SP, Wood GA (1972). Catamenial pneumothorax. JAMA..

[5] Bobbio A, Carbognani P, Ampollini L, Rusca M (2007). Diaphragmatic laceration, partial liver herniation and catamenial pneumothorax. Asian Cardiovasc Thorac Ann.

[6] Bostoen S, Van Raemdonck D, Dooms C (2011). Why a chest physician should be interested in abdominal pain. Acta Clin Belg.

[7] Sanna S, Taurchini M, Monteverde M, Agnoletti V, Casoni GL (2011). Catamenially recurring pneumothorax with partial liver herniation: a particular view. Respiration..

[8] Tomescot A, Fabre D (2012). Catamenial pneumothorax with multiple transdiaphragmatic hepatic herniations. Asian Cardiovasc Thorac Ann.

[9] Yu PS, Sihoe AD (2015). Beware the ‘raised right hemidiaphragm’ in a female patient with previous pneumothorax surgery: liver herniation through a massive endometrosis-related diaphragmatic fenestration. Thorac Dis.

[10] Wong I, Afzal A, Gulkarov I, Chang R, Reyes A, Worku B (2017). Recurrent pneumothoraces: making the link to catamenial pneumothorax. Am J Med.

[11] Demetrio L, Francisco S, Hernán B, Javier C, Lidia D, Iván R (2018). Thoracic and diaphragmatic endometriosis: single-institution experience using novel, broadened diagnostic criteria. J Turk Ger Gynecol Assoc.

[12] Mukku VK, Cassidy E, Negulescu C, Jagneaux T, Godke J (2019). Large spontaneous right catamenial pneumothorax with diaphragmatic defect and liver herniation. Case Rep Pulmonol.

[13] Larraín D, Suárez F, Braun H, Chapochnick J, Diaz L, Rojas I (2018). Thoracic and diaphragmatic endometriosis: single-institution experience using novel, broadened diagnostic criteria. J Turk Ger Gynecol Assoc..

